# Analysis of Chemical Constituents in Wuzi-Yanzong-Wan by UPLC-ESI-LTQ-Orbitrap-MS

**DOI:** 10.3390/molecules201219765

**Published:** 2015-12-01

**Authors:** Dixin Zou, Jinfeng Wang, Bo Zhang, Suhua Xie, Qing Wang, Kexin Xu, Ruichao Lin

**Affiliations:** 1 Beijing Key Lab of Chinese Materia Medica Quality Evaluation, School of Chinese Pharmacy, Beijing University of Chinese Medicine, Beijing 100102, China; zoudixin@163.com (D.Z.); qing_wang_123@126.com (J.W.); 13301080686@163.com (B.Z.); xiaogangvsmylove@163.com (Q.W.); 18547789137@163.com (K.X.); 2 Beijing Tong Ren Tang Group Co., Ltd., Beijing 100051, China; 15301127363@163.com

**Keywords:** Wuzi-Yanzong-Wan, UPLC-ESI-LTQ-Orbitrap-MS, constituents, identification

## Abstract

Wuzi-Yanzong-Wan (WZYZW), a classical traditional Chinese medicine (TCM) prescription containing *Fructus Lych*, *Semen Cuscutae* (fried), *Fructus Rubi*, *Fructus* *Schisandrae* *chinensis* (steamed) and *Semen Plantaginis* (fried with salt), is widely used to treat impotence, sterility, spermatorrhea, premature ejaculation, lumbago and post-micturation dribble. However, the chemical profile of WZYZW has not been established yet. In this work, a rapid and sensitive method for systematically screening and identifying the chemical constituents of WZYZW in both positive and negative ion modes using Ultra-Performance LC coupled with ESI-linear ion trap-Orbitrap tandem mass spectrometry (UPLC-ESI-LTQ-Orbitrap-MS) has been developed. Based on the chromatographic and spectrometric data, and referring to the literature, we could tentatively identify 106 compounds, including organic acids, flavonoids, phenylpropanoids, alkaloids and terpenoids. Fourteen ingredients from *Fructus* *Lych* were identified, while 10 ingredients were from *Semen Cuscutae* (fried), 33 ingredients were from *Fructus Rubi*, 37 ingredients were from *Fructus Schisandrae chinensis* (steamed), and 20 ingredients were from *Semen Plantaginis* (fried with salt). The results may provide essential data for further quality control, pharmacological research and clinical evaluation of WZYZW. Furthermore, this study indicates the developed approach based on UPLC-ESI-LTQ-Orbitrap-MS is suitable for characterizing the chemical profiles of TCM prescriptions. This is the first report to provide a comprehensive analysis of the chemical constituents of WZYZW.

## 1. Introduction

Chinese herbal prescriptions, the basic form of clinical application of TCM for thousands of years, have been proven by clinical practice to play a positive role in human health. The multi-chemical constituents of Chinese herbal prescriptions are the key to achieving their prevention and treatment effects, so comprehensive analysis of their chemical constituents is the premise and foundation to promote development and innovation in TCM [1,2,3]. Therefore, it is necessary to develop rapid and sensitive methods for identifying the chemical constituents of TCM prescriptions, to facilitate their quality control, safe and effective use in clinic.

Wuzi-Yanzong-Wan (WZYZW), the most classical Chinese herb prescription for nourishing the kidney and strengthening the essence, is called “ancient and modern seed first choice prescription”, being firstly detailed recorded in She-sheng-zhong-miao-fang treatise of the Ming Dynasty and widely used to treat syndrome of kidney deficiency and damage of essence, including impotence, sterility, spermatorrhea, premature ejaculation, lumbago and post-micturation dribble [4,5,6]. WZYZW is composed of *Fructus Lych*, *Semen Cuscutae* (fried), *Fructus Rubi*, *Fructus Schi-sandrae chinensis* (steamed), and *Semen Plantaginis* (fried with salt).

Over the years, the chemical work on WZYZW has mainly focused on either qualitative or quantitative analyses of its major components using different techniques [7,8,9,10], such as HPLC, LC-MS, *etc.* These methods have contributed to the study of WZYZW to some extent, but there has been no systematic characterization of the chemical constituents in this prescription, which could contribute to interpret the material basis for its therapeutic effects.

The comprehensive analysis of the chemical constituents in Chinese herbal prescriptions and herbal medicines is hard work, on account of the fact they typically include extraordinarily complex components. LTQ-Orbitrap-MS as a high resolution mass spectrometry technique has proven to be an advanced, accurate and reliable tool for the comprehensive identification of compounds in complex systems such as TCMs [11,12,13,14,15]. It has high sensitivity, resolution and mass accuracy, which provides multi-stage MS analysis (MS^n^) information, acquisition of precursor ion data of high-resolution, determination of accurate mass charge ratios, structural information of fragment ions from MS^n^, and gives the elementary composition of compounds and allows the structure confirmation of trace components in complicated samples [16]. A C18 column with a 1.7 µm particle pore size is employed for UPLC, resulting in higher peak capacity, greater resolution, and faster and more sensitive separations in comparison with HPLC [17,18]. Therefore, the present study is aimed at establishing a rapid and sensitive method for making the constituents of WZYZW more clear and comprehensible, using the UPLC-LTQ-Orbitrap-MS technique in both negative and positive modes. As a result, a total of 106 constituents were screened and identified, and the developed method was proved efficient, giving accurate and information-rich results. To our knowledge, this is the first time a comprehensive analysis of the multiple class compounds of WZYZW has been reported.

## 2. Results and Discussion

### 2.1. Optimization of Analytical Conditions

To obtain better chromatographic separation and mass spectrometric detection, four mobile phase systems: methanol-aqueous solution, acetonitrile-aqueous solution, acetonitrile-formic acid aqueous solution and acetonitrile containing formic acid-formic acid aqueous solution were examined, the acetonitrile containing formic acid-formic acid aqueous solution showed a better degree of separation. Formic acid (0.1%) was added to the mobile phase to enhance the intensity of mass signals and improve the peak shape. In addition, the flow rate (0.25, 0.3, 0.35 mL/min), column temperature (25, 30, 40 °C) and injection volume (2, 3, 5 uL) were respectively optimized in this work. The chromatographic separation was better when the chromatographic conditions were as follows: acetonitrile containing formic acid-aqueous formic acid solution, flow rate of 0.3 mL/min, column temperature of 30 °C and injection volume of 3 μL.

### 2.2. Chemical Constituents Identification in WZYZW

**Figure 1:**
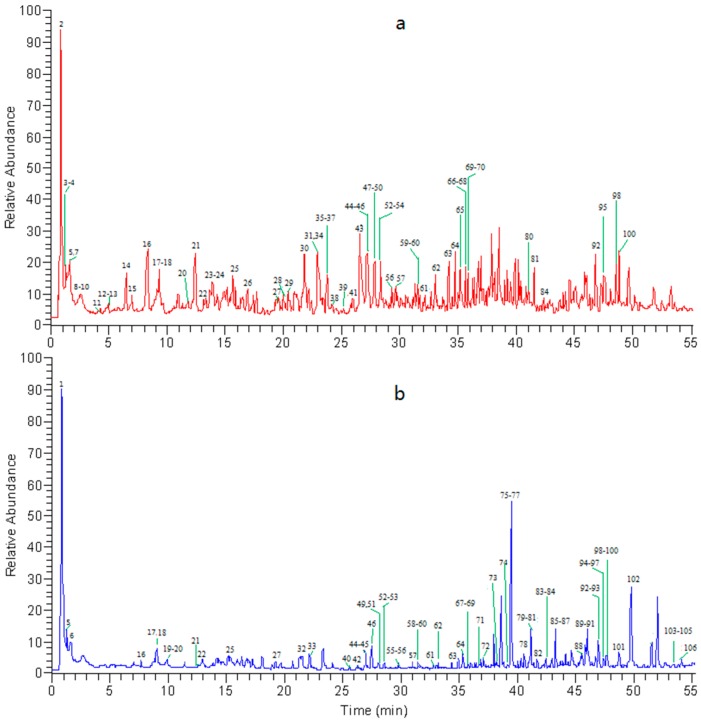
Total ion chromatogram (TIC) of WZYZW in negative ion mode (**a**) and positive ion mode (**b**) using UPLC-ESI-LTQ-Orbitrap-MS.

Chemical profiles of WZYZW in both negative and positive modes were well separated and detected by using the established UPLC-ESI-LTQ-Orbitrap-MS method. The TIC chromatograms in both ESI modes are shown in Figure 1.

In this study, for the compounds with available standards, the compound was identified by comparing the retention time and high-resolution accurate mass with the reference compound. Thus peaks 1, 5, 6, 10, 14, 17, 19, 20, 21, 27, 36, 51, 59, 60, 61, 64, 65, 68 and 75 were unambiguously identified as abromine, nicotinic acid, thiamine, riboflavin, taurine, quinic acid, atropine, ferulic acid, chlorogenic acid, scopoletin, rutin, esculin, apigenin, hesperidin, quercetin, kaempferol, luteolin, isorhamnetin and schisandrin, respectively. Meanwhile, the fragmentation patterns and pathways of the standards helped further confirm the structures of the derivatives of the reference compounds. When no standard compounds were available, the structures were elucidated by adopting the following measures to improve the quality of the identifications: firstly, based on the high-accuracy precursor ions obtained from the LTQ-Orbitrap-MS, using Xcalibur 2.1, the element compositions were calculated following some rules (C = 40, H = 100, O = 30, N = 5, Na = 2 and RDB equivalent value = 15). The maximum tolerance of mass error for all the precursor and product ions was set at 5 ppm, which can satisfy the requirements for positive identification. Besides, the most rational molecular formula was sought in different chemical databases such as the Spectral Database for Organic Compounds SDBS (http://sdbs.db.aist.go.jp) and Massbank (http://www.massbank.jp). When several matching compounds with the same formula were found, the structures were sought in the chemical information of the compounds from the five crude drugs of WZYZW. The component herb from which each compound was derived was confirmed by individually analyzing the herbs with the same UPLC-LTQ-Orbitrap-MS method. Finally, the chemical structures were further confirmed by combining the chromatographic data, the high resolution and accurate mass product ion information provided by data-dependent scan from MS^n^ experiments and referring to literature references. Moreover, the fragmentation patterns and pathways of the standards contributed to further confirming the derivatives of the reference compounds, then, the structure could be tentatively identified. A total of 106 compounds of WZYYW were thus identified or tentatively identified, including 35 flavonoids, 34 phenylpropanoids, 17 organic acids, 8 alkaloids, 11 terpenoids and one miscellaneous ingredient; 14 ingredients from *Fructus Lych* were identified, 10 ingredients from *Semen Cuscutae* (fried), 33 ingredients from *Fructus Rubi*, 37 ingredients from *Fructus Schisandrae chinensis* (steamed), and 20 ingredients from *Semen Plantaginis* (fried with salt). Table 1 summarizes their information.

**Table 1:** Identification of the chemical constituents of WZYZW by UPLC-ESI-LTQ-Obitrap-MS.

No.	tR (min)	Molecular Formula	Caculated Mass (*m*/*z*)	Experimental Mass (*m*/*z*)		MS/MS Fragments		Identification Compound	Source	
				N-ion	ppm	P-ion	ppm			
1	0.81	C_5_H_11_NO_2_	117.08626	―	―	118.08569	−0.6	235[2M + H]^+^;	Abromine	a
								118[M + H]^+^;		
								59[C_3_H_9_N]^+^;		
								58[C_3_H_8_N]^+^		
2	0.87	C_6_H_8_O_6_	176.02371	175.02289	−4.7	―	―	175[M − H]^−^;	Ascorbic acid	a
								115[C_4_H_3_O_4_]^−^;		
								113[C_5_H_5_O_3_]^−^		
3	1.12	C_6_H_8_O_7_	192.01863	191.0189	1.4	―	―	191[M − H]^−^;	Citric acid	d
								173[M − H − H_2_O]^−^;		
								129[M − H − H_2_O − CO_2_]^−^;		
								111[M − H − H_2_O − COOH − OH]^−^		
4	1.12	C_4_H_6_O_5_	134.01315	133.01372	4.3	―	―	133[M + H]^+^;	Malic acid	d
								115[M + H − H_2_O]^+^		
5	1.28	C_6_H_5_NO_2_	123.03931	122.02417	4.2	124.03876	−4.3	122[M − H]^−^;	Nicotinic acid	a
								124[M + H]^+^;		
								106 [M + H − H_2_O]^+^		
6	1.58	C_12_H_16_N_4_OS	264.11176	―	―	265.11071	−3.9	265[M + H]^+^;	Thiamine	a
								156[C_7_H_10_NOS]^+^;		
								144[C_6_H_10_NOS]^+^;		
								122[C_6_H_8_N_3_]^+^		
7	1.97	C_4_H_6_O_4_	118.01824	117.01791	−2.8	―	―	117[M − H]^−^;	Succinic acid	d
								99[M − H − H_2_O]^−^;		
								73[M − H − CO_2_]^−^		
8	2.68	C_7_H_6_O_5_	170.01315	169.01301	−0.8	―	―	169[M − H]^−^;	Gallic acid	c
								125[M **–** H − CO_2_]^−^		
9	3.08	C_4_H_6_O_6_	150.00806	149.00808	0.1	―	―	149[M − H]^−^;	Tartaric acid	d
								131[M − H − H_2_O]^−^;		
								103[M − H − HCOOH]^−^		
10	3.28	C_17_H_20_N_4_O_6_	376.12991	375.12988	−0.1	―	―	375[M − H]^−^;	Riboflavin	a
								255[C_13_H_11_N_4_O_2_]^−^;		
								212[C_12_H_10_N_3_O]^−^		
11	4.48	C_7_H_6_O_3_	138.02332	137.02365	2.4	―	―	137[M − H]^−^;	Salicylic acid/	c
								93[C_6_H_5_O]^−^	4-Hydroxyben-zoic acid	
12	4.66	C_7_H_6_O_4_	154.01824	153.01862	2.5	―	―	153[M − H]^−^;	Protocatechuic acid	d
								109[M − H − CO_2_]^−^		
13	4.74	C_7_H_6_O_3_	138.02332	137.02373	3	―	―	137[M − H]^−^;	Salicylic acid/	c
								93[C_6_H_5_O]^−^	4-Hydroxyben-zoic acid	
14	7.2	C_2_H_7_NO_3_S	125.00629	124.00638	0.7	―	―	124[M − H]^−^;	Taurine	a
								80[SO_3_]^−^		
15	7.38	C_8_H_8_O_4_	168.03389	167.0342	1.9	―	―	167[M − H]^−^;	Vanillic acid	a,c
								152[M − H − CH_3_]^−^;		
								123[M − H − CO_2_]^−^;		
								108[M − H − CH_3_ − CO_2_]^−^		
16	8.35	C_16_H_22_O_10_	374.11293	373.11377	2.3	397.10892	−4.0	373[M − H]^−^;	Geniposidic acid	e
								329[M − H − CO_2_]^−^;		
								211[M − H − Glc]^−^;		
								193 [M − H − Glc − H_2_O]^−^;		
								167[M − H − Glc − CO_2_]^−^;		
								149[M − H − Glc − CO_2_ − H_2_O]^−^;		
								397[M + Na]^+^;		
								353[M + Na − CO_2_]^+^;		
								235[M + Na − Glc]^+^;		
								217[M + Na − Glc − H_2_O]^+^;		
								149[C_7_H_10_O_2_Na]^+^		
17	8.79	C_7_H_12_O_6_	192.05502	191.05503	0.1	193.06981	−4.4	191[M − H]^−^;	Quinic acid	d
								173[M − H − H_2_O]^−^;		
								155[M − H − 2H_2_O]^−^;		
								127[M − H − 2H_2_O − CO]^−^;		
								193 [M + H]^+^;		
								175[M + H − H_2_O]^+^;		
								157[M + H − 2H_2_O]^+^		
18	9.68	C_9_H_8_O_4_	180.03389	179.03429	2.2	181.04889	−3.5	179[M − H]^−^;	Caffeic acid	a
								135[M − H − CO_2_]^−^;		
								181[M + H]^+^;		
								163[M + H − H_2_O]^+^;		
								145[M + H − 2H_2_O]^+^		
19	10.2	C_17_H_23_NO_3_	289.17507	―	―	290.17578	2.4	290[M + H]^+^;	Atropine	a
								272[M + H − H_2_O]^+^;		
								260[M + H − HCHO]^+^;		
								242[M + H − HCHO − H_2_O]^+^;		
								124[C_8_H_14_N]^+^;		
								95[C_7_H_11_]^+^		
20	11.91	C_10_H_10_O_4_	194.04954	193.05001	2.4	195.06447	−3.7	193[M − H]^−^;	Ferulic Acid	a,c
								175[M − H − H_2_O]^−^;		
								149[M − H − CO_2_]^−^;		
								134[M − H − CH_3_ − CO_2_]^−^;		
								195[M + H]^+^;		
								177[M + H − H_2_O]^+^;		
								145[M + H − H_2_O − CH_3_ − OH]^+^		
21	12.37	C_16_H_18_O_9_	354.08671	353.08731	1.7	355.10097	−3.9	353[M − H]^−^;	Chlorogenic acid	a
								191[C_7_H_11_O_6_]^−^;		
								173[C_7_H_9_O_5_]^−^;		
								127[C_6_H_7_O_3_]^−^;		
								355[M + H]^+^;		
								163[C_9_H_7_O_3_]^+^;		
								117[C_8_H_5_O]^+^		
22	13.1	C_9_H_8_O_3_	164.03897	163.03905	0.5	165.05393	−4.2	163[M − H]^−^;	*p*-Coumaric acid	a
								119[M − H − CO_2_]^−^;		
								165 [M + H]^+^;		
								147[M + H − H_2_O]^+^		
23	13.64	C_7_H_10_O_5_	174.04445	173.04381	−3.7	―	―	173[M − H]^−^;	Shikimic acid	c
								155[M − H − H_2_O]^−^;		
								137[M − H − 2H_2_O]^−^;		
								128[M − H − COOH]^−^		
24	13.97	C_27_H_30_O_17_	626.24908	625.25148	3.8	―	―	625[M − H]^−^;	Quercetin-3-*O*-β-d-galactopyra-noside-7-*O*-β-d-glucopyranoside	b
								463[M − H − Gal]^−^;		
								301[M − H − Gal − Glc]^−^;		
								300[M − H − Gal − Glc − H]^−^;		
								273[M − H − Gal − Glc − CO]^−^;		
								255[M − H − Gal − Glc − CO − H_2_O]^−^		
25	15.6	C_9_H_6_O_4_	178.01824	177.01847	1.3	179.03317	−4.0	177[M − H]^−^;	Esculetin	c
								149[M − H − CO]^−^;		
								133[M − H − CO_2_]^−^;		
								121[M − H − 2CO]^−^;		
								105[M − H − CO_2_ − CO]^−^;		
								93[M − H − 3CO]^−^;		
								179[M + H]^+^;		
								151[M + H − CO]^+^;		
								133[M + H − CO − H_2_O]^+^		
26	17.17	C_22_H_22_O_11_	462.10784	461.10962	3.9	―	―	461[M − H]^−^;	Homoplantagini-n	e
								299[M − H − Glu]^−^;		
								271[M − H − Glu − CO]^−^;		
								181[C_8_H_5_O_5_]^−^		
27	19.43	C_10_H_8_O_4_	192.03387	191.03419	1.6	193.04933	−1.1	191[M − H]^−^;	Scopoletin	a
								193[M + H]^+^;		
								178[M + H − CH_3_]^+^;		
								165[M + H − CO]^+^;		
								161[M + H − CH_3_OH]^+^;		
								133[M + H − CH_3_OH − CO]^+^		
28	19.6	C_21_H_20_O_12_	464.08711	463.08829	2.6	―	―	463[M − H]^−^;	6-Hydroxy-luteolin-7-*O*-glucoside	e
								301[M − H − Glc]^−^;		
								273[M − H − Glc − CO]^−^;		
								257[M − H − Glc − CO_2_]^−^;		
								167[C_7_H_3_O_5_]^−^		
29	20.93	C_22_H_20_O_12_	476.0871	475.08633	−1.6	―	―	475[M − H]^−^;	Kaempferol-3-*O*-β-d-glucuronic acid methyl ester	c
								457[M − H − H_2_O]^−^;		
								433[M − H − CO=CH_2_]^−^;		
								415[M − H − CH_3_COOH]^−^;		
								285[M − H − C_7_H_10_O_6_]^−^		
30	21.62	C_15_H_10_O_7_	302.03428	301.03396	−1.1	―	―	301[M − H]^−^;	6-Hydroxy-luteolin	e
								273[M − H − CO]^−^;		
								257[M − H − CO_2_]^−^;		
								167[C_7_H_3_O_5_]^−^		
31	21,78	C_26_H_28_O_16_	596.14463	595.14543	1.4	―	―	595[M − H]^−^;	Quercetin-3-*O*-β-d-galactosyl-(1→2)-β-d-apioside	b
								505[M − H – Api + C_2_H_2_O]^−^;		
								463[M − H − Api]^−^;		
								445[M − H − Api − H_2_O]^−^;		
								301[M − H − Api − Gal]^−^;		
								300[M − H − Api − Gal − H]^−^;		
								273[M − H − Api − Gal − CO]^−^;		
								257[M − H − Api − Gal − 2CO]^−^;		
								179[C_8_H_3_O_5_]^−^		
32	21.88	C_14_H_6_O_8_	302.01355	―	―	303.01321	−1.1	303[M+H]^+^;	Ellagic acid	c
								285[M + H − H_2_O]^+^;		
								275[M + H − CO]^+^;		
								257[M + H − CO − H_2_O]^+^;		
								247[M + H − 2CO]^+^;		
								201[C_11_H_5_O_4_]^+^		
33	22.26	C_29_H_28_O_9_	520.18061	―	―	521.18042	−0.4	521[M + H]^+^;	Schisantherin D	d
								399[M + H − C_6_H_5_COOH]^+^;		
								355[M + H − C_6_H_5_COOH − C_2_H_4_O]^+^		
34	22.9	C_21_H_20_O_12_	464.08711	463.08887	3.8	―	―	463[M − H]^−^;	Hyperoside	b,c
								301[M − H − Gal]^−^;		
								255[M − H − Gal − CO − H_2_O]^−^;		
								151[C_7_H_3_O_4_]^−^;		
								107[C_6_H_3_O_2_]^−^		
35	23.02	C_16_H_12_O_6_	300.05502	299.05453	−1.6	―	―	299[M − H]^−^;	Hispidulin	e
								271[M − H − CO]^−^;		
								255[M − H − CO_2_]^−^;		
								227[M − H − CO − CO_2_]^−^;		
								181[C_8_H_5_O_5_]^−^		
36	23.1	C_27_H_30_O_16_	610.14501	609.1479	4.7	―	―	609[M − H]^−^;	Rutin	c
								343[C_17_H_11_O_8_]^−^;		
								301[M − H − Rha − Glc]^−^;		
								300[M − H − Rha − Glc − H]^−^;		
								271[M − H − Rha − Glc − H − CO − H]^−^;		
								255[M − H − Rha − Glc − H − CO − OH]^−^;		
								179[C_8_H_3_O_5_]^−^;		
								151[C_7_H_3_O_4_]^−^		
37	23.74	C_21_H_20_O_12_	464.08711	463.0874	0.6	―	―	463[M − H]^−^;	Isoquercitrin	c
								301[M − H − Glc]^−^;		
								255[M − H − Glc − CO − H_2_O]^−^;		
								151[C_7_H_3_O_4_]^−^;		
								107[C_6_H_3_O_2_]^−^		
38	24.2	C_29_H_36_O_16_	640.19197	639.19263	1	―	―	639[M − H]^−^;	Plantamajoside	e
								477[M − H − C_9_H_6_O_3_]^−^;		
								315[M − H − C_9_H_6_O_3_ − Glc]^−^;		
								153[M − H − C_9_H_6_O_3_ − 2Glc]^−^;		
								135[M − H − C_9_H_6_O_3_ − 2Glc − H_2_O]^−^		
39	24.95	C_27_H_30_O_15_	594.15009	593.1507	1	―	―	593[M − H]^−^;	Kaempferol-3-*O*-rutinoside	c
								327[M − H − C_10_H_18_O_8_]^−^;		
								285[M − H − Rha − Glc]^−^;		
								284[M − H − Rha − Glc − H]^−^;		
								257[M − H − Rha − Glc − CO]^−^;		
								255[M − H − Rha − Glc − H − CO − H]^−^;		
								229[M − H − Rha − Glc − 2CO]^−^;		
								227[M − H − Rha − Glc − H − 2CO − H]^−^		
40	25.34	C_28_H_36_O_8_	500.24829	―	―	501.25002	3.4	501[M + H]^+^;	Tigloylgomisin H/Angeloygomi-sin H	d
								484[M + H − OH]^+^;		
								483[M + H − H_2_O]^+^;		
								384[M + H − OH − C_4_H_7_COOH]^+^;		
								357[M + H − C_4_H_7_COOH − C_2_H_4_O]^+^		
41	25.95	C_21_H_20_O_11_	448.09219	447.09378	3.6	―	―	447[M − H]^−^;	Luteoloside	e
								285[M − H − Glc]^−^;		
								284[M − H − Glc − H]^−^;		
								257[M − H − Glc − CO]^−^;		
								243[M − H − Glc − C_2_H_2_O]^−^;		
								241[M − H − Glc − CO_2_]^−^;		
								199[M − H − Glc − C_2_H_2_O − CO_2_]^−^;		
								197[M − H − Glc − 2CO_2_]^−^;		
								151[C_7_H_3_O_4_]^−^		
42	26.1	C_28_H_36_O_8_	500.24829	―	―	501.25071	4.8	501[M + H]^+^;	Tigloylgomisin H/Angeloygomi-sin H	d
								484[M + H − OH]^+^;		
								483[M + H − H_2_O]^+^;		
								384[M + H − OH − C_4_H_7_COOH]^+^;		
								357[M + H − C_4_H_7_COOH − C_2_H_4_O]^+^		
43	26.57	C_29_H_36_O_15_	624.19707	623.19604	−1.6	―	―	623[M − H]^−^;	Acteoside	e
								461[M − H − C_9_H_6_O_3_]^−^;		
								315[M − H − C_9_H_6_O_3_ − Rha]^−^;		
								179[C_9_H_7_O_4_]^−^;		
								153[M − H − C_9_H_6_O_3_ − Rha − Glc]^−^;		
								135[M − H − C_9_H_6_O_3_ − Rha − Glc − H_2_O]^−^		
44	27.19	C_21_H_20_O_11_	448.09219	447.09225	0.1	449.10532	−2.5	447[M − H]^−^;	Astragalin	b,c
								285[M − H − Glc]^−^;		
								151[C_7_H_3_O_4_]^−^;		
								449[M + H]^+^;		
								287[M − H − Glc]^+^;		
								241[M − H − Glc − CH_2_O_2_]^+^;		
								213[M + H − Glc − C_2_H_2_O_3_]^+^		
45	27.19	C_21_H_20_O_11_	448.09219	447.09225	0.1	449.10699	−1.9	447[M − H]^−^;	Plantaginin	e
								285[M − H − Glc]^−^;		
								267[M − H − Glc − H_2_O]^−^;		
								255[M − H − Glc − CH_2_O]^−^;		
								239[M − H − Glc − H_2_O − CO]^−^;		
								167[C_7_H_3_O_5_]^−^;		
								449[M + H]^+^;		
								287[M+H − Glc]^+^;		
								269[M + H − Glc − H_2_O]^+^;		
								169[C_7_H_5_O_5_]^+^		
46	27.48	C_29_H_36_O_15_	624.19705	623.19794	1.4	625.21005	−4.2	623[M − H]^−^;	Methylhesperidin	c
								477[M − H − Rha]^−^;		
								315[M − H − Rha − Glc]^−^;		
								300[M − H − Rha − Glc − CH_3_]^−^;		
								297[M − H − Rha − Gldc − H_2_O]^−^;		
								285[M − H − Rha − Glc − 2CH_3_]^−^;		
								282[M − H − Rha − Glc − H_2_O − CH_3_]^−^;		
								272[M − H − Rha − Glc − CH_3_ − CO]^−^;		
								257[M − H − Rha − Glc − 2CH_3_ − CO]^−^;		
								229[M − H − Rha − Glc − 2CH_3_ − 2CO]^−^;		
								625[M + H]^+^;		
								479[M + H − Rha]^+^;		
								317[M + H − Rha − Glc]^+^;		
								299[M + H − Rha − Glc − H_2_O]^+^;		
								281[M + H − Rha − Glc − 2H_2_O]^+^;		
								193[C_10_H_9_O_4_]^+^;		
								165[C_9_H_9_O_3_]^+^		
47	27.72	C_21_H_20_O_11_	448.09219	447.09402	4.1	―	―	447[M − H]^−^;	Quercitrin	c
								301[M − H − Rha]^−^;		
								300[M − H − Rha − H]^−^;		
								273[M − H − Rha − CO]^−^;		
								255[M − H − Rha − CO − H_2_O]^−^		
48	27.86	C_22_H_22_O_12_	478.10275	477.10461	3.9	―	―	477[M − H]^−^;	Nepetin-7-*O*-glucoside	e
								315[M − H − Glc]^−^;		
								287[M − H − Glc − CO]^−^;		
								271[M − H − Glc − CO_2_]^−^;		
								181[C_8_H_5_O_5_]^−^		
49	27.93	C_21_H_20_O_10_	432.09727	431.09805	1.8	433.11093	−4.6	431[M − H]^−^;	Kaempferol-7-*O*-α-l-rhamnoside	c
								413[M − H − H_2_O]^−^;		
								285[M − H − Rha]^−^;		
								267[M − H − Rha − H_2_O]^−^;		
								169[C_12_H_9_O]^−^;		
								155[C_11_H_7_O]^−^;		
								433[M + H]^+^		
50	27.97	C_21_H_20_O_10_	432.09727	431.09866	3.2			431[M − H]^−^;	Apigenin-7-*O*-glucoside	e
								269[M − H − Glc]^−^;		
								201[M − H − Glc − C_3_O_2_]^−^;		
								151[C_7_H_3_O_4_]^−^		
51	27.99	C_15_H_16_O_9_	340.07106	339.07058	−1.4	―	―	339[M − H]^−^;	Esculin	c
								177[M − H − Glc]^−^;		
								133[M − H − Glc − CO_2_]^−^;		
								105[M − H − Glc − CO_2_ − CO]^−^;		
								89[C_7_H_5_]^−^		
52	28.28	C_27_H_30_O_14_	578.15518	577.15662	2.5	579.17073	−0.2	577[M − H]^−^;	Rhoifolin	e
								457[M − H − C_4_H_8_O_4_]^−^;		
								431[M − H − Rha]^−^;		
								413[M − H − Rha − H_2_O]^−^;		
								269[M − H − Rha − Glc]^−^;		
								225[M − H − Rha − Glc − CO_2_]^−^;		
								183[C_12_H_7_O_2_]^−^;		
								579[M + H]^+^;		
								433[M + H − Rha]^+^;		
								415[M + H − Rha − H_2_O]^+^;		
								397[M + H − Rha − 2H_2_O]^+^;		
								271[M + H − Rha − Glc]^+^;		
								253[M + H − Rha − Glc − H_2_O]^+^;		
								243[M + H − Rha − Glc − CO]^+^;		
								225[M + H − Rha − Glc − H_2_O − CO]^+^;		
								211[M + H − Rha − Glc − H_2_O − C_2_H_2_O]^+^;		
								153[C_7_H_5_O_4_]^+^;		
								119[C_8_H_7_O]^+^;		
								91[C_7_H_7_]^+^		
53	28.28	C_28_H_32_O_16_	624.16066	623.16248	2.9	625.17896	4.2	623[M − H]^−^;	Isorhamnetin-3-*O*-β-d-rutinoside	a
								477[M − H − Rha]^−^;		
								315[M − H − Rha − Glc]^−^;		
								300[M − H − Rha − Glc − CH_3_]^−^;		
								272[M − H − Rha − Glc − CH_3_ − CO]^−^;		
								271[M − H − Rha − Glc − CH_3_ − CO − H]^−^;		
								243[M − H − Rha − Glc − CH_3_ − CO − H − CO]^−^;		
								227[M − H − Rha − Glc − CH_3_ − CO − H − CO_2_]^−^;		
								151[C_7_H_3_O_4_]^−^;		
								625[M + H]^+^;		
								479[M + H − Rha]^+^;		
								317[M + H − Rha − Glc]^+^;		
								302[M + H − Rha − Glc − CH_3_]^+^;		
								285[M + H − Rha − Glc − CH_3_OH]^+^;		
								274[M + H − Rha − Glc − CH_3_ − CO]^+^;		
								257[M + H − Rha − Glc − CH_3_OH − CO]^+^;		
								246[M + H − Rha − Glc − CH_3_ − 2CO]^+^;		
								229[M + H − Rha − Glc − CH_3_OH − 2CO]^+^;		
								153[C_7_H_5_O_4_]^+^		
54	28.31	C_21_H_18_O_11_	446.07654	445.07493	−3.6	―	―	445[M − H]^−^;	Baicalin	e
								269[M − H − GluA]^−^;		
								225[M − H − GluA − CO_2_]^−^;		
								167[C_7_H_3_O_5_]^−^		
55	29.09	C_20_H_26_O_4_	330.19039	―	―	331.18914	−3.8	331[M + H]^+^;	*Meso*-dihydrog-uaiaretic acid	d
								313[M + H − H_2_O]^+^;		
								301[M + H − 2CH_3_]^+^		
56	29.58	C_15_H_10_O_5_	270.0601	269.09566	−1.6	271.05966	−1.7	269[M − H]^−^;	Baicalein	e
								241[M − H − CO]^−^;		
								225[M − H − CO_2_]^−^;		
								197[M − H − CO − CO_2_]^−^;		
								167[C_7_H_3_O_5_]^−^;		
								271[M + H]^+^;		
								253[M + H − H_2_O]^+^;		
								169[C_7_H_5_O_5_]^+^		
57	30.7	C_28_H_34_O_15_	610.1814	609.18229	1.5	611.1994	3.9	609[M − H]^−^;	Neohesperidin	c
								505[M − H − C_4_H_8_O_3_]^−^;		
								463[M − H − Rha]^−^;		
								445[M − H − Rha − H_2_O]^−^;		
								427[M − H − Rha − 2H_2_O]^−^;		
								301[M − H − Rha − Glc]^−^;		
								286[M − H − Rha − Glc − CH_3_]^−^;		
								283[M − H − Rha − Glc − H_2_O]^−^;		
								268[M − H − Rha − Glc − H_2_O − CH_3_]^−^;		
								258[M − H − Rha − Glc − CH_3_ − CO]^−^;		
								257[M − H − Rha − Glc − CO_2_]^−^;		
								242[M − H − Rha − Glc − CH_3_ − CO_2_]^−^;		
								239[M − H − Rha − Glc − H_2_O − CO_2_]^−^;		
								199[C_12_H_7_O_3_]^−^;		
								125[C_6_H_5_O_3_]^−^;		
								611[M + H]^+^;		
								303[M + H − Rha − Glc]^+^;		
								285[M + H − Rha − Glc − H_2_O]^+^;		
								179[C_9_H_7_O_4_]^+^;		
								177[C_10_H_9_O_3_]^+^;		
								151[C_8_H_7_O_3_]^+^		
58	31.48	C_23_H_28_O_8_	432.1857	―	―	433.18538	−0.7	433[M + H]^+^;	Schizandradiol	d
								415[M + H − H_2_O]^+^;		
								361[M + H − C_4_H_8_O]^+^;		
								372[M + H − H_2_O − CH_3_CO]^+^;		
								343[M + H − H_2_O − C_4_H_7_OH]^+^		
59	31.54 39.44	C_15_H_10_O_5_	270.04445	269.04353	−3.4	271.06145	4.9	269[M − H]^−^;	Apigenin	e
								241[M − H − CO]^−^;		
								227[M − H − C_2_H_2_O]^−^;		
								225[M − H − CO_2_]^−^;		
								201[M − H − C_3_O_2_]^−^;		
								151[C_7_H_3_O_4_]^−^;		
								271[M + H]^+^;		
								253[M + H − H_2_O]^+^;		
								225[M + H − H_2_O − CO]^+^		
60	31.71	C_28_H_34_O_15_	610.1814	609.18351	3.5	611.20001	4.8	609[M − H]^−^;	Hesperidin	c
								463[M − H − Rha]^−^;		
								301[M − H − Rha − Glc]^−^;		
								286[M − H − Rha − Glc − CH_3_]^−^;		
								283[M − H − Rha − Glc − H_2_O]^−^;		
								268[M − H − Rha − Glc − H_2_O − CH_3_]^−^;		
								258[M − H − Rha − Glc − CH_3_ − CO]^−^;		
								257[M − H − Rha − Glc − CO_2_]^−^;		
								242[M − H − Rha − Glc − CH_3_ − CO_2_]^−^;		
								239[M − H − Rha − Glc − H_2_O − CO_2_]^−^;		
								199[C_12_H_7_O_3_]^−^;		
								125[C_6_H_5_O_3_]^−^;		
								611[M + H]^+^;		
								303[M + H − Rha − Glc]^+^;		
								285[M + H − Rha − Glc − H_2_O]^+^;		
								179[C_9_H_7_O_4_]^+^;		
								177[C_10_H_9_O_3_]^+^;		
								151[C_8_H_7_O_3_]^+^		
61	32.13	C_15_H_10_O_7_	302.03428	301.03549	4	303.0488	−1.1	301[M − H]^−^;	Quercetin	b,c
								273[M − H − CO]^−^;		
								255[M − H − CO − H_2_O]^−^;		
								151[C_7_H_3_O_4_]^−^;		
								303[M + H]^+^;		
								285[M + H − H_2_O]^+^;		
								257[M + H − H_2_O − CO]^+^;		
								247[M + H − 2CO]^+^;		
								229[M + H − H_2_O − 2CO]^+^		
62	33.02	C_30_H_26_O_13_	594.12897	593.13049	2.6	595.14142	−3.2	593[M − H]^−^;	Tiliroside	c
								447[M − H − C_9_H_6_O_2_]^−^;		
								285[M − H − C_9_H_6_O_2_ − Glc]^−^;		
								257[M − H − C_9_H_6_O_2_ − Glc − CO]^−^;		
								229[M − H − C_9_H_6_O_2_ − Glc − 2CO]^−^;		
								151[C_7_H_3_O_4_]^−^;		
								617[M + Na]^+^;		
								595[M + H]^+^;		
								449[M + H − C_9_H_6_O_2_]^+^;		
								287[M + H − C_9_H_6_O_2_ − Glc]^+^		
63	34.12	C_15_H_12_O_6_	288.05502	287.05549	1.7	289.07002	−2.2	287[M − H]^−^;	Aromadendrin	c
								269[M − H − H_2_O]^−^;		
								259[M − H − CO]^−^;		
								243[M − H − CO_2_]^−^;		
								225[M − H − H_2_O − CO_2_]^−^;		
								215[M − H − CO − CO_2_]^−^;		
								151[C_7_H_3_O_4_]^−^;		
								107[C_6_H_3_O_2_]^−^;		
								289[M + H]^+^;		
								271[M + H − H_2_O]^+^;		
								195[C_9_H_7_O_5_]^+^;		
								145[C_9_H_5_O_2_]^+^		
64	35.18	C_15_H_10_O_6_	286.03936	285.03995	2.1	287.05359	4.9	285[M − H]^−^;	Kaempferol	b,c
								257[M − H − CO]^−^;		
								229[M − H − 2CO]^−^;		
								151[C_7_H_3_O_4_]^−^;		
								309[M + Na]^+^;		
								287[M + H]^+^;		
								241[M + H − CH_2_O_2_]^+^;		
								213[M + H − C_2_H_2_O_3_]^+^		
65	35.31	C_15_H_10_O_6_	286.03936	285.04019	2.9	―	―	571[2M − H]^−^;	Iuteolin	e
								285[M − H]^−^;		
								243[M − H − C_2_H_2_O]^−^;		
								241[M − H − CO_2_]^−^;		
								199[M − H − C_2_H_2_O − CO_2_]^−^;		
								197[M − H − 2CO_2_]^−^;		
								171[M − H − C_2_H_2_O − CO_2_ − CO]^−^;		
								151[C_7_H_3_O_4_]^−^		
66	35.58	C_16_H_12_O_7_	316.04993	315.05117	3.9	―	―	315[M − H]^−^;	Nepetin	e
								287[M − H − CO]^−^;		
								271[M − H − CO_2_]^−^;		
								243[M − H − CO − CO_2_]^−^;		
								181[C_8_H_5_O_5_]^−^		
67	35.61	C_15_H_10_O_6_	286.03937	285.03989	1.8	287.05627	4.4	285[M − H]^−^;	Scutellarein	e
								257[M − H − CO]^−^;		
								241[M − H − CO_2_]^−^;		
								167[C_7_H_3_O_5_]^−^;		
								287[M + H]^+^;		
								269[M + H − H_2_O]^+^;		
								241[M + H − H_2_O − CO]^+^;		
								169[C_7_H_5_O_5_]^+^		
68	35.61	C_16_H_12_O_7_	316.04993	315.05027	1.1	317.06486	−2.3	315[M − H]^−^;	Isorhamnetin	b
								300[M − H − CH_3_]^−^;		
								272[M − H − CH_3_ − CO]^−^;		
								271[M − H − CH_3_ − CO − H]^−^;		
								243[M − H − CH_3_ − CO − H − CO]^−^;		
								227[M − H − CH_3_ − CO − H − CO_2_]^−^;		
								151[C_7_H_3_O_4_]^−^;		
								107[C_6_H_3_O_2_]^−^;		
								339[M + Na]^+^;		
								317[M + H]^+^;		
								302[M + H − CH_3_]^+^;		
								285[M + H − CH_3_OH]^+^;		
								274[M + H − CH_3_ − CO]^+^;		
								257[M + H − CH_3_OH − CO]^+^;		
								246[M + H − CH_3_ − 2CO]^+^;		
								229[M + H − CH_3_OH − 2CO]^+^;		
								153[C_7_H_5_O_4_]^+^		
69	35.76	C_30_H_46_O_4_	470.34689	469.33251	2.7	471.34509	−3.8	469[M − H]^−^;	Nigranoic acid	d
								423[M − H − HCOOH]^−^;		
								378[M − H − HCOOH − HCOO]^−^;		
								471[M + H]^+^;		
								456[M + H − CH_3_]^+^;		
								453[M + H − H_2_O]^+^;		
								390[M + H − C_6_H_9_]^+^		
70	35.89	C_28_H_24_O_16_	616.09807	615.10097	4.7	―	―	615[M − H]^−^;	2″-*O*-Galloylhyperoside	c
								463[M − H − C_7_H_4_O_4_]^−^;		
								445[M − H − C_7_H_6_O_5_]^−^;		
								301[M − H − C_7_H_4_O_4_ − Glc]^−^		
71	36.79	C_23_H_30_O_7_	418.20643	―	―	419.20477	−4.0	419[M + H]^+^;	Gomisin H	d
								401[M + H − H_2_O]^+^;		
								384[M + H − OH − H_2_O]^+^;		
								383[M + H − 2H_2_O]^+^;		
								369[M + H − OH − CH_3_ − H_2_O]^+^;		
								353[M + H − OH − OCH_3_ − H_2_O]^+^		
72	37.08	C_28_H_34_O_8_	498.23264	―	―	499.23119	−2.9	499[M + H]^+^;	Angeloygomisin O/Angeloylisogomisin O	d
								399[M + H − C_4_H_7_COOH]^+^;		
								369[M + H − C_4_H_7_COOH − CH_2_O]^+^;		
								357[M + H − C_4_H_7_COOH − C_3_H_6_]^+^;		
								343[M + H − C_4_H_7_COOH − C_4_H_8_]^+^		
73	38.12	C_28_H_34_O_8_	498.23264	―	―	499.23026	−4.8	499[M + H]^+^;	Angeloygomisin O/Angeloylisogomisin O	d
								399[M + H − C_4_H_7_COOH]^+^;		
								369[M + H − C_4_H_7_COOH − CH_2_O]^+^;		
								357[M + H − C_4_H_7_COOH − C_3_H_6_]^+^;		
								343[M + H − C_4_H_7_COOH − C_4_H_8_]^+^		
74	39.28	C_23_H_28_O_7_	416.19078	―	―	417.18903	−4.2	417[M + H]^+^;	Gomisin O/Epigomisin O	d
								402[M + H − CH_3_]^+^;		
								399[M + H − H_2_O]^+^;		
								385[M + H − CH_3_OH]^+^;		
								373[M + H − C_2_H_4_O]^+^		
75	39.38	C_24_H_32_O_7_	432.22209	―	―	433.22103	−2.4	433[M + H]^+^;	Schisandrin	d
								415[M + H − H_2_O]^+^;		
								400[M + H − H_2_O − CH_3_]^+^;		
								384[M + H − H_2_O − OCH_3_]^+^;		
								373[M + H − H_2_O − C_3_H_6_]^+^;		
								359[M + H − H_2_O − C_4_H_8_]^+^		
76	39.44	C_30_H_46_O_4_	470.34689	―	―	471.34551	−2.9	471[M + H]^+^;	Kadsuric acid	d
								453[M + H − H_2_O]^+^;		
								398[M + H − CH_2_CH_2_COOH]^+^;		
								370[M + H − CH_2_CH_2_COOH − C_2_H_4_]^+^		
77	39.6	C_23_H_28_O_7_	416.19078	―	―	417.19103	0.6	417[M + H]^+^;	Gomisin O/Epigomisin O	d
								402[M + H − CH_3_]^+^;		
								399[M + H − H_2_O]^+^;		
								385[M + H − CH_3_OH]^+^;		
								373[M + H − C_2_H_4_O]^+^		
78	40.63	C_28_H_34_O_10_	530.22247	―	―	531.22218	−0.6	531[M + H]^+^;	Gomisin D	d
								485[M + H − CH_2_O_2_]^+^;		
								401[M + H − C_6_H_10_O_3_]^+^;		
								383[M + H − C_6_H_10_O_3_ − H_2_O]^+^		
79	40.98	C_22_H_28_O_6_	388.19586	―	―	389.19503	−2.1	389[M + H]^+^;	Gomisin J	d
								374[M + H − CH_3_]^+^;		
								357[M + H − CH_3_OH]^+^;		
								342[M + H − CH_3_OH − CH_3_]^+^		
80	41.02	C_30_H_48_O_5_	488.3418	487.3439	4.3	489.35526	−4.5	487[M − H]^−^;	Rosolic acid/2α,3α,19α-Trihydroxyolean-12-ene-28-oic acid/Arjunolic acid	c
								469[M − H − H_2_O]^−^;		
								443[M − H − CO_2_]^−^;		
								425[M − H − CO_2_ − H_2_O]^−^;		
								407[M − H − CO_2_ − 2H_2_O]^−^;		
								391[M − H − CO_2_ − 2H_2_O − CH_4_]^−^;		
								489[M + H]^+^;		
								453[M + H − 2H_2_O]^+^		
81	41.22	C_30_H_48_O_5_	488.3418	487.34378	4	489.35544	−4.1	487[M − H]^−^;	Rosolic acid/2α,3α,19α-Trihydroxyolean-12-ene-28-oic acid/Arjunolic acid	c
								469[M − H − H_2_O]^−^;		
								443[M − H − CO_2_]^−^;		
								425[M − H − CO_2_ − H_2_O]^−^;		
								407[M − H − CO_2_ − 2H_2_O]^−^;		
								391[M − H − CO_2_ − 2H_2_O − CH_4_]^−^;		
								489[M + H]^+^;		
								453[M + H − 2H_2_O]^+^		
82	41.75	C_15_H_24_N_2_O_2_	264.10889	―	―	265.18997	−4.0	265[M + H]^+^;	Sophoranol	b
								248[M + H − OH]^+^;		
								247[M + H − H_2_O]^+^		
83	42.18	C_30_H_46_O_3_	454.35198	―	―	455.35025	−3.8	455[M + H]^+^;	Ganwuweizic acid	d
								409[M + H − HCOOH]^+^;		
								313[M + H − C_8_H_14_O_2_]^+^		
84	42.32	C_30_H_48_O_5_	488.3418	487.34302	2.5	489.35529	−4.4	487[M − H]^−^;	Rosolic acid/2α,3α,19α-Trihydroxyolean-12-ene-28-oic acid/Arjunolic acid	c
								469[M − H − H_2_O]^−^;		
								443[M − H − CO_2_]^−^;		
								425[M − H − CO_2_ − H_2_O]^−^;		
								407[M − H − CO_2_ − 2H_2_O]^−^;		
								391[M − H − CO_2_ − 2H_2_O − CH_4_]^−^;		
								489[M + H]^+^;		
								453[M + H − 2H_2_O]^+^		
85	42.89	C_12_H_16_N_2_O	204.13354	―	―	205.13409	2.6	205[M + H]^+^;	*N*-methylcytisine	b
								146[M + H − C_3_H_9_N]^+^;		
								108[C_6_H_6_NO]^+^		
86	43.27	C_23_H_28_O_6_	400.19587	―	―	401.19434	−3.8	401[M + H]^+^;	Schizandrin B	d
								386[M + H − CH_3_]^+^;		
								370[M + H − OCH_3_]^+^;		
								355[M + H − CH_3_ − OCH_3_]^+^;		
								339[M + H − 2CH_3_ − CH_3_OH]^+^		
87	43.3	C_30_H_34_O_8_	522.23265	―	―	523.23095	−3.2	523[M + H]^+^;	Benzoylgomisin H	d
								505[M + H − H_2_O]^+^;		
								401[M + H − C_6_H_5_COOH]^+^;		
								383[M + H − C_6_H_5_COOH − H_2_O]^+^		
88	45.42	C_23_H_30_O_6_	402.21151	―	―	403.21011	−3.5	403[M + H]^+^;	Schisanhenol	d
								372[M + H − OCH_3_]^+^;		
								371[M + H − CH_3_OH]^+^;		
								356[M + H − CH_3_OH − CH_3_]^+^;		
								340[M + H − OCH_3_ − CH_3_OH]^+^		
89	45.44	C_23_H_28_O_7_	416.19078	―	―	417.18957	−2.9	417[M + H]^+^;	Schisandrol B	d
								399[M + H − H_2_O]^+^;		
								343[M + H − C_4_H_8_ − H_2_O]^+^;		
								307[M + H − 2CH_3_ − 2OCH_3_ − H_2_O]^+^		
90	45.67	C_28_H_34_O_9_	514.22755	―	―	515.22559	−3.8	515[M + H]^+^;	Schisantherin B/Schisantherin C	d
								415[M + H − C_4_H_7_COOH]^+^;		
								385[M + H − C_4_H_7_COOH − CH_2_O]^+^;		
								367[M + H − C_4_H_7_COOH − CH_2_O − H_2_O]^+^;		
								353[M + H − C_4_H_7_COOH − CH_2_O − CH_3_OH]^+^		
91	46.06	C_30_H_32_O_9_	536.21191	―	―	537.20981	−3.9	537[M + H]^+^;	Schisantherin A	d
								415[M + H − C_6_H_5_COOH]^+^;		
								397[M + H − C_6_H_5_COOH − H_2_O]^+^;		
								373[M + H − C_6_H_5_COOH − C_3_H_6_]^+^		
92	46.58	C_30_H_48_O_4_	472.34689	471.34872	3.9	473.30911	−3.7	471[M − H]^−^;	Corosolic acid/Maslinic acid	c
								453[M − H − H_2_O]^−^;		
								427[M − H − CO_2_]^−^;		
								391[M − H − CO_2_ − 2H_2_O]^−^;		
								473[M + H]^+^;		
								455[M + H − H_2_O]^+^;		
								203[C_15_H_23_]^+^;		
								105[C_8_H_9_]^+^		
93	46.73	C_15_H_24_N_2_O	248.19614	―	―	249.19699	2.2	271[M + Na]^+^;	Matrine	b
								249[M+H]^+^;		
								231[M + H − H_2_O]^+^;		
								150[C_10_H_16_N]^+^;		
								148[C_10_H_14_N]^+^		
94	47.28	C_22_H_26_O_6_	386.18022	―	―	387.17992	−0.8	387[M + H]^+^;	Gomisin L_1_/Gomisin L_2_/Gomisin M_1_/Gomisin M_2_	d
								372[M + H − CH_3_]^+^;		
								369[M + H − H_2_O]^+^;		
								357[M + H − CH_2_O]^+^;		
								329[M + H − C_2_H_2_O_2_]^+^		
95	47.52	C_30_H_48_O_4_	472.34689	471.34863	3.7	473.36044	−4.4	471[M − H]^−^;	Corosolic acid/Maslinic acid	c
								453[M − H − H_2_O]^−^;		
								427[M − H − CO_2_]^−^;		
								391[M − H − CO_2_ − 2H_2_O]^−^;		
								473[M + H]^+^;		
								455[M + H − H_2_O]^+^;		
								203[C_15_H_23_]^+^;		
								105[C_8_H_9_]^+^		
96	47.53	C_22_H_26_O_6_	386.18022	―	―	387.17847	−4.5	387[M + H]^+^;	Gomisin L_1_/Gomisin L_2_/Gomisin M_1_/Gomisin M_2_	d
								372[M + H − CH_3_]^+^;		
								369[M + H − H_2_O]^+^;		
								357[M + H − CH_2_O]^+^;		
								329[M + H − C_2_H_2_O_2_]^+^			
97	47.76	C_28_H_34_O_9_	514.22755	―	―	515.22716	−0.7	515[M + H]^+^;	Schisantherin B/Schisantherin C	d
								415[M + H − C_4_H_7_COOH]^+^;		
								385[M + H − C_4_H_7_COOH − CH_2_O]^+^;		
								367[M + H − C_4_H_7_COOH − CH_2_O − H_2_O]^+^;		
								353[M + H − C_4_H_7_COOH − CH_2_O − CH_3_OH]^+^		
98	48.01	C_30_H_48_O_3_	456.36763	455.35424	4.9	457.36636	−2.7	455[M − H]^−^;	Oleanolic acid/Ursolic acid	c,e
								407[M − H − HCHO − H_2_O]^−^;		
								391[M − H − HCHO − H_2_O − CH_4_]^−^;		
								479[M + Na]^+^;		
								457[M + H]^+^;		
								439[M + H − H_2_O]^+^;		
								393[M + H − HCOOH − H_2_O]^+^		
99	48.24	C_22_H_26_O_6_	386.18022	―	―	387.17883	−3.6	387[M + H]^+^;	Gomisin L_1_/Gomisin L_2_/Gomisin M_1_/Gomisin M_2_	d
								372[M + H − CH_3_]^+^;		
								369[M + H − H_2_O]^+^;		
								357[M + H − CH_2_O]^+^;		
								329[M + H − C_2_H_2_O_2_]^+^		
100	48.4	C_30_H_48_O_3_	456.36763	455.35394	4.3	457.36612	−3.3	455[M − H]^−^;	Oleanolic acid/Ursolic acid	c,e
								407[M − H − HCHO − H_2_O]^−^;		
								391[M − H − HCHO − H_2_O − CH_4_]^−^;		
								479[M + Na]^+^;		
								457[M + H]^+^;		
								457[M + H − H_2_O]^+^;		
								393[M + H − HCOOH − H_2_O]^+^		
101	48.65	C_22_H_26_O_6_	386.18022	―	―	387.17877	−3.7	387[M + H]^+^;	Gomisin L_1_/Gomisin L_2_/Gomisin M_1_/Gomisin M_2_	d
								372[M + H − CH_3_]^+^;		
								369[M + H − H_2_O]^+^;		
								357[M + H − CH_2_O]^+^;		
								329[M + H − C_2_H_2_O_2_]^+^			
102	49.79	C_24_H_32_O_6_	416.22714	―	―	417.22649	−1.6	417[M + H]^+^;	Deoxyschizan-drin	d
								402[M + H − CH_3_]^+^;		
								386[M + H − OCH_3_]^+^;		
								370[M + H − CH_3_ − CH_3_OH]^+^;		
								347[M + H − CH_3_ − C_4_H_7_]^+^;		
								316[M + H − C_6_H_13_O]^+^;		
								286[M + H − C_6_H_13_O − 2CH_3_]^+^;		
								273[M + H − C_6_H_13_O − CH_3_ − CO]^+^;		
								227[M + H − C_6_H_13_O − CO − OCH_3_ − 2CH_3_]^+^		
103	52.02	C_16_H_14_O_4_	270.09649	―	―	271.09595	−2.0	271[M + H]^+^; 203[M + H − C_5_H_8_]^+^;	Imperatorin	c
								175[M + H − C_5_H_8_ − CO]^+^;		
								159[M + H − C_5_H_8_ − CO_2_]^+^		
104	53.29	C_30_H_32_O_8_	520.21701	―	―	521.21559	−2.7	521[M + H]^+^;	Benzoylgomisin O/Benzoyisolgomisin O	d
								399[M + H − C_6_H_5_COOH]^+^;		
								369[M + H − C_6_H_5_COOH − CH_2_O]^+^;		
								357[M + H − C_6_H_5_COOH − C_3_H_6_]^+^;		
								343 M + H − C_6_H_5_COOH − C_4_H_8_]^+^		
105	53.43	C_22_H_24_O_6_	384.16457	―	―	385.16406	−1.3	385[M + H]^+^;	Schizandrin C	d
								370[M + H − CH_3_]^+^;		
								355[M + H − CH_2_O]^+^		
106	54.08	C_30_H_32_O_8_	520.21701	―	―	521.21611	−1.7	521[M + H]^+^;	Benzoylgomisin O/Benzoyisolgomisin O	d
								399[M + H − C_6_H_5_COOH]^+^;		
								369[M + H − C_6_H_5_COOH − CH_2_O]^+^;		
								357[M + H − C_6_H_5_COOH − C_3_H_6_]^+^;		
								343 M + H − C_6_H_5_COOH − C_4_H_8_]^+^		

a: *Fructus Lych*, b: *Semen Cuscutae* (fried), c: *Fructus Rubi*, d: *Fructus Schisandrae chinensis* (steamed), e: *Semen Plantaginis* (fried with salt); Glc : β-d-glucose, GluA: Glucuronic acid, Xyl: β-d-xylose, Rha: l-rhamnose; Gal: d-galactose; Api: d-apiose, ppm: difference between calculated and found mass.

#### 2.2.1. Flavonoids

**Figure 2:**
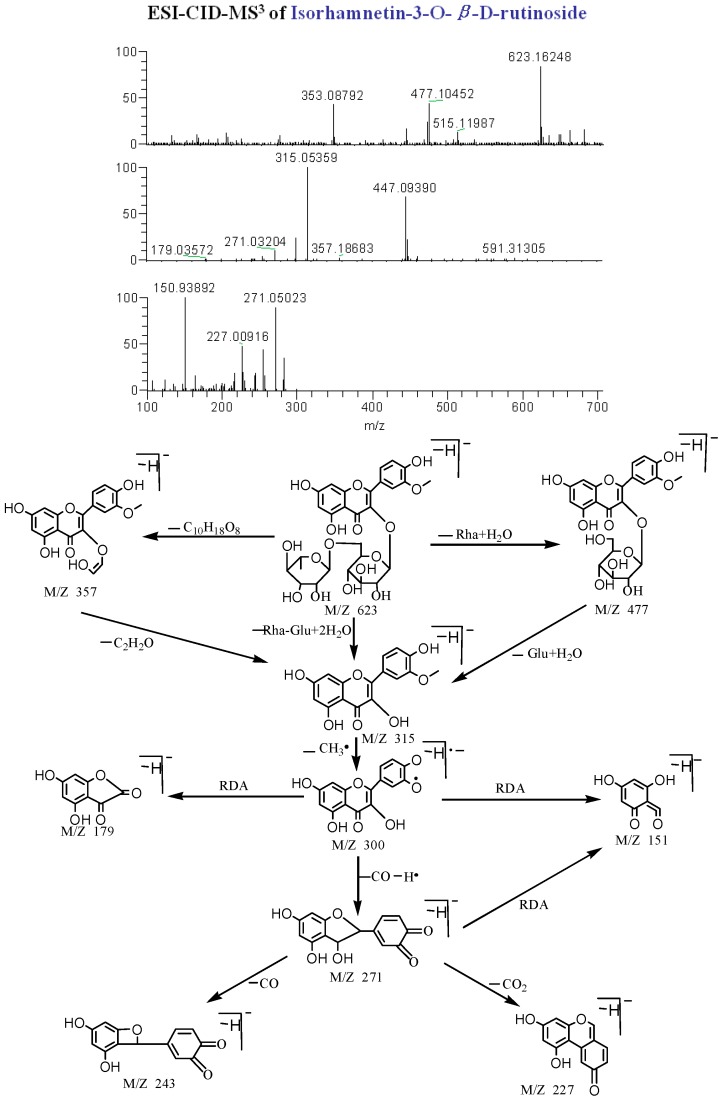
ESI-CID-MS^3^ spectra and proposed fragmentations for isorhamnetin-3-*O*-β-d-rutinoside (**53**) from WZYZW.

Flavonoids have a diphenylpropane skeleton bearing two benzene rings (A and B) connected by a pyran ring attached to the A ring, and are further divided into several subclasses (flavones, flavonols, flavanones, flavanonols, anthocyanidins, aurone, halcones and isoflavonoids). In this work, four types of flavonoids were found in WZYZW by UPLC–ESI-LTQ-Orbitrap-MS. Taking compound **53** as an example, the precise molecular weight is 623.16248 (C_28_H_31_O_16_) and in the negative ion spectrum, the main fragment ions were observed at *m/z* 623 [M − H]^−^, 477 [M − H − Rha]^−^, 315 [M − H − Rha − Glc]^−^, 300 [M − H − Rha − Glc − CH_3_]^−^, 272 [M − H − Rha − Glc − CH_3_ − CO]^−^, 271 [M − H − Rha − Glc − CH_3_ − CO − H]^−^, 243 [M − H − Rha − Glc − CH_3_ − CO − H − CO]^−^, 227 [M − H − Rha − Glc − CH_3_ − CO − H − CO_2_]^−^, and 151 [C_7_H_3_O_4_]^−^, thus, compound **53** was identified as isorhamnetin-3-*O*-β-d-rutinoside. Its mass spectrum and proposed fragmentation pathways in negative mode are shown in Figure 2.

Compounds **34** and **37** were considered to be isomers as they displayed the same [M − H]^−^ ions at *m/z* 463.08887 (C_21_H_19_O_12_) and 463.08740 (C_21_H_19_O_12_). Moreover, they had the similar fragment ions of *m/z* 301, 255 and 151 in the MS^n^ spectra, but different max UV absorption wavelengths (λ_max_), as the λ_max_ of compound **34** were 254 and 356 nm, and compound **37** showed λ_max_ peaks at 254 and 346 nm. Considering the different retention times, compounds **34** and **37** were tentatively identified as hyperoside and isoquercitrin, respectively (see Table 1).

**Figure 3:**
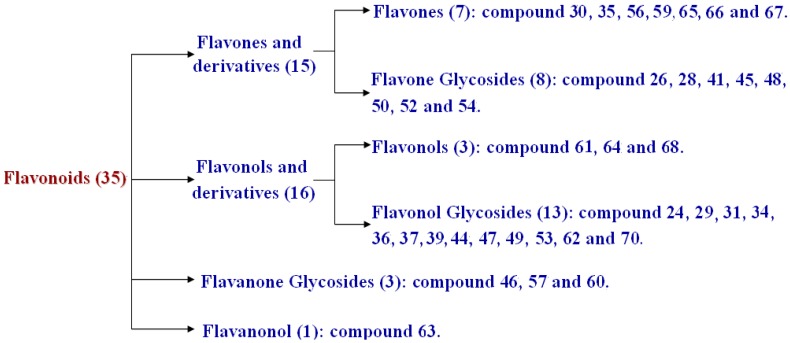
Flavonoids identified in WZYZW by UPLC-ESI-LTQ-Orbitrap-MS.

Similarly, based on the chromatographic behavior and MS^n^ spectrometry for further confirming the fragmentation patterns as shown above, a total of 35 flavonoids were tentatively identified, including 15 flavones and derivatives, 16 flavonols and derivatives, three flavanone glycosides, and one flavanonol, which are summarized in Figure 3.

#### 2.2.2. Phenylpropanoids

**Figure 4:**
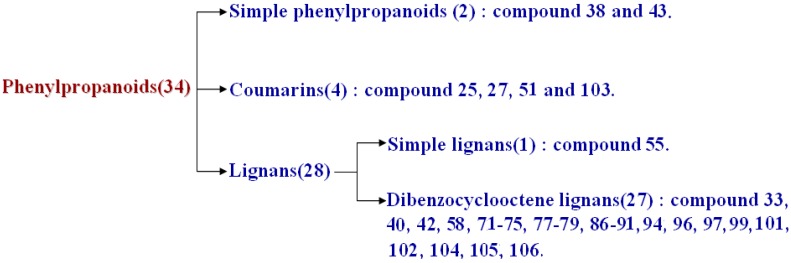
Phenylpropanoids identified in WZYZW by UPLC-ESI-LTQ-Orbitrap-MS.

Phenylpropanoids have one or more C_6_-C_3_ structures and there are three main subclasses: simple phenylpropanoids, coumarins and lignans. In the present study, 34 phenylpropanoids were found in WZYZW, including two simple phenylpropanoids, four coumarins and 28 lignans. The classification of these compounds is shown in Figure 4.

**Figure 5:**
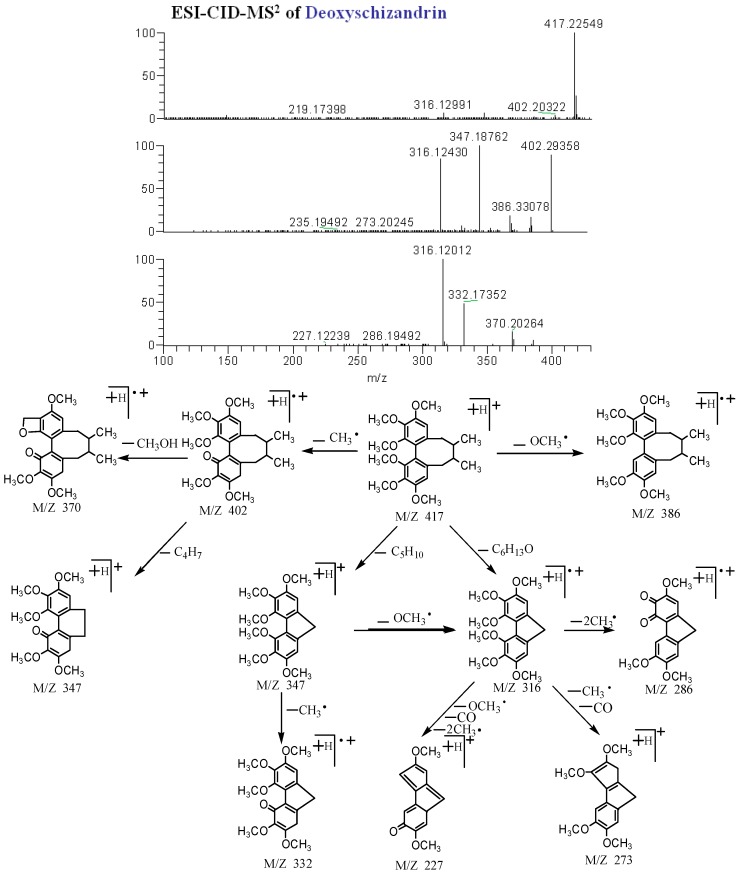
ESI-CID-MS^3^ spectra and proposed fragmentations for deoxyschizandrin (**102**) from WZYZW.

Compound **102** afforded a quasi-molecular ion at *m/z* 417.22649 (C_24_H_3__3_O_6_) in positive mode, and its MS^n^ spectra showed representative ions at *m/z* 417 [M + H]^+^, 402 [M + H − CH_3_]^+^, 386 [M + H − OCH_3_]^+.^, 370 [M + H − CH_3_ − CH_3_OH]^+^, 347 [M + H − CH_3_ − C_4_H_7_]^+^, 316 [M + H − C_6_H_13_O]^+^, 286 [M + H − C_6_H_13_O − 2CH_3_]^+^, 273 [M + H − C_6_H_13_O − CH_3_ − CO]^+^, and 227 [M + H − C_6_H_13_O – CO − OCH_3_ − 2CH_3_]^+^. Consequently, compound **102** was tentatively identified as deoxyschizandrin. The MS^n^ mass spectra and the fragmentation pathways of deoxyschizandrin are shown in Figure 5. As with the fragmentations described above, the other phenylpropanoid compounds were also tentatively identified. However, six groups of isomers (compounds **40** and **42**, **72** and **73**, **74** and **77**, **90** and **97**, **94**, **96**, **99** and **101**, **104** and **106**) had the same molecular weights in the MS spectra, similar fragment ions in the MS^n^ spectra and similar max UV absorption wavelengths. Thus we could not distinguish them, and these structures will require further confirmation (see Table 1).

#### 2.2.3. Organic Acids

**Figure 6:**
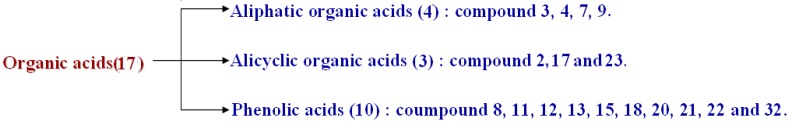
Organic acids identified in WZYZW by UPLC-ESI-LTQ-Orbitrap-MS.

The UPLC-ESI-LTQ-Orbitrap method was applied to comprehensively characterize the organic acids in this study, and the results showed that three types of organic acids could be detected in WZYZW: four aliphatic organic acids, three alicyclic organic acids and 10 phenolic acids. The specific details are displayed in Figure 6.

Compound **15** produced a [M − H]^−^ ion at *m/z* 167.03420 (C_8_H_7_O_4_), and its fragment ions showed the losses of CH_3_, CO_2_ and CH_3_ + CO_2_, respectively. Thus, compound **15** was identified as vanillic acid, which was confirmed by MS^n^ experiments. Similarly, the above fragmentation patterns were applied to further confirm the identities of the other organic acids compound, and finally, 17 compounds were identified as organic acids.

#### 2.2.4. Terpenoids

**Figure 7:**
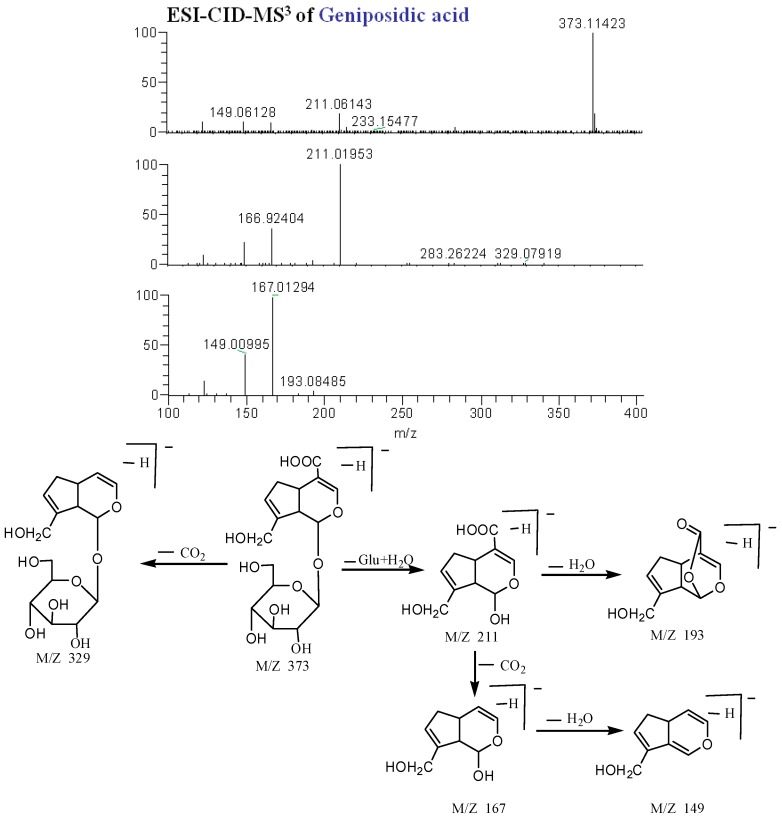
ESI-CID-MS^3^ spectra and proposed fragmentations for geniposidic acid (**16**) from WZYZW.

As displayed in Figure 7, the precise molecular weight of compound **16** is 373.11377 (C_16_H_2__1_O_10_) in the negative ion spectrum, and the main fragment ions were observed at *m/z* 373 [M − H]^−^, 329 [M – H − CO_2_]^−^, 211 [M − H − Glc]^−^, 193 [M − H − Glc − H_2_O]^−^, 167 [M − H − Glc − CO_2_]^−^ and 149 [M − H − Glc − CO_2_ − H_2_O]^−^. Therefore, compound **16** was tentatively identified as geniposidic acid.

Using high-resolution MS^n^ mass spectrometry and the similar method of analysis of the aforementioned fragmentations, a total of 11 terpenoids were found: one iridoid (compound **16**) was identified as geniposidic acid; 10 triterpenes—compounds **69**, **76**, **80**, **81**, **83**, **84**, **92**, **95**, **98** and **100** were identified as nigranoic acid, kadsuric acid, rosolic acid, 2α,3α,19α-trihydroxyolean-12-ene-28-oic acid, ganwuweizic acid, arjunolic acid, corosolic acid, maslinic acid, oleanolic acid and ursolic acid. Compounds **80**, **81** and **84**, **98** and **100**, and **92** and **95**, constituted three groups of isomers with similar fragmentation pathways in their MS spectra, so we could not precisely identify them by Mass (see Table 1).

#### 2.2.5. Alkaloids

Compound **5** exhibited a [M + H]^+^ ion and a [M − H]^−^ at *m/z* 124.03876 (C_6_H_6_NO_2_) and 122.02417 (C_6_H_4_NO_2_), respectively, and positive product ions at *m/z* 124 [M + H]^+^ and 106 [M + H − H_2_O]^+^ were detected. Thus, compound **5** was inferred as nicotinic acid (see Table 1). Similarly, eight alkaloids were identified in WZYZW and confirmed using MS^n^ data: abromine (**1**), nicotinic acid (**5**), thiamine (**6**), taurine (**14**), atropine (**19**), sophoranol (**82**), *n*-methylcytisine (**85**), and matrine (**93**).

#### 2.2.6. Miscellaneous

Riboflavin (compound **10**) was also identified in WZYZW by its [M − H]^−^ ion at *m/z* 375.12988 (C_17_H_19_N_4_O_6_). The corresponding MS^n^ spectra showed a peak at *m/z* 255 [C_13_H_11_N_4_O_2_]^−^, and another fragment ion was also observed at *m/z* 212 [C_12_H_10_ N_3_O]^−^.

To summarize, in this study, a reliable and rapid UPLC-ESI-LTQ-Orbitrap-MS method has been established for the first comprehensive analysis of the phytochemical constituents of the Chinese herbal prescription WZYZW. This method revealed that UPLC-ESI-LTQ-Orbitrap-MS was useful for screening and identifying the complex constituents of WZYZW. Based on the method, a total of 106 compounds were tentatively characterized. In contrast to the published papers, it is noteworthy that this work detected more components. Our results provide essential data for further pharmacological studies and clinical evaluation of WZYZW and be useful for quality control of WZYZW, so as to guarantee its safe use in the clinic.

## 3. Experimental Section

### 3.1. Materials and Reagents

*Fructus Lych*, *Semen Cuscutae* (fried), *Fructus Rubi*, *Fructus Schisandrae chinensis* (steamed), and *Semen Plantaginis* (fried with salt) were supplied by the Tongrentang Drug Store in Beijing (China). The origins of the crude drugs were identified by Professor Rui Chao Lin of the Department of Pharmacognosy, Beijing University of Chinese Medicine. Voucher specimens were deposited at the authors’ laboratory. Reference standards of abromine, nicotinic acid, thiamine, riboflavin, taurine, quinic acid, atropine, ferulic acid, chlorogenic acid, scopoletin, rutin, esculin, apigenin, hesperidin, quercetin, kaempferol, luteolin, isorhamnetin, schisandrin were purchased from the National Institute for the Control of Pharmaceutical and Biological Products (Beijing, China). Acetonitrile and methanol (HPLC grade) were purchased from Fisher Scientific Co. (Waltham, MA, USA). Distilled water was purchased from Watson’s Food & Beverage Co. (Guangzhou, China). Formic acid, analytical grade, was obtained from Beijing Reagent Company (Beijing, China). Other reagents were HPLC grade or analytical reagent grade. High-purity nitrogen (99.9%) and helium (99.99%) were purchased from Gas Supplies Center of Peking University Health Science Center (Beijing, China).

### 3.2. Preparation of WZYZW Samples

A standard preparation of WZYZW was made in accordance with the 2015 version of the Chinese Pharmacopoeia. *Fructus Lych*, *Semen Cuscutae* (fried), *Fructus Rubi*, *Fructus Schisandrae chinensis* (steamed), and *Semen Plantaginis* (fried with salt) (8:8:4:2:1) were weighed, then crushed to powder (40 mesh size) and immersed in 10-fold volume of water for 1 h and heated. After boiling, heating was ontinued until the volume was reduced by 5-fold. The mixture was filtered through gauze while hot, concentrated to 1 g crude drug/mL, and freeze-dried to a powder. Finally refined honey (85 g) was mixed with freeze-dried powder (100 g) to make boluses of WZZYW for the experiments. Freeze-dried single herb extract powders were prepared as described for WZYZW. Freeze-dried samples of WZYZW (250 mg) were extracted with 50 mL of methanol with the aid of ultrasound for 60 min. The extracts were centrifuged at 10,000 rpm for 15 min at 4 °C, the supernatant was collected and filtered through a filter (0.22 μm), and the filtrate was collected for UPLC-LTQ-Orbitrap analysis. Respective standard stock solutions of 19 components were prepared at concentrations of 50 ng/mL by weighing the desired amount of each component into a volumetric flask and dissolving it in 100% ethanol; the 19 samples were filtered through a filter (0.22 μm), and the filtrates were analyzed by UPLC-LTQ-Orbitrap.

### 3.3. Instrumentation and Conditions

#### 3.3.1. Liquid Chromatography Conditions

The chromatographic separation was performed on an ACQUITY UPLC^TM^ BEH C_18_ column (1.7 μm, 2.1 mm × 100 mm) using an ACQUITY UPLC^TM^ system (Waters Corporation, Milford, MA, USA) equipped with quaternary pump, vacuum degasser, autosampler and photodiode array detector. A linear gradient elution of A (HCOOH:H_2_O = 0.1:100) and B (HCOOH:CH_3_CN = 0.1:100) was used. The optimized gradient program is shown in Table 2. The flow rate was 0.3 mL/min and column temperature was set at 30 °C, the injection volume was 3 μL. The effluent was roughly split at a ratio of 3:1 (*v*/*v*) before entering the ESI source.

#### 3.3.2. ESI-MS/MS Detection

The LTQ/Orbitrap mass spectrometer (Thermo Scientific, Bremen, Germany) was equipped with an ESI source operating in positive and negative ESI mode. The negative ion mode operation parameters were as follows: capillary voltage, 35 V; electrospray voltage, 3.0 kV; capillary temperature, 350 °C; sheath gas, 30 (arbitrary units); auxiliary gas, 10 (arbitrary units); tube lens, 110 V. The positive ion mode operation parameters: capillary voltage, 25 V; electrospray voltage, 4.0 kV; capillary temperature, 350 °C; sheath gas, 30 (arbitrary units); auxiliary gas, 5(arbitrary units ); tube lens, 110 V. Samples were detected by full-scan mass analysis from *m*/*z* 100 to 1000 at a resolving power of 30,000 with data-dependent MS^2^ analysis triggered by the three most-abundant ions from the predicted precursor list followed by MS^3^ analysis of the most-abundant product ions. To avoid performing many repeated data acquisitions on the same sample, dynamic exclusion is used for the data collection with an exclusion duration of 60s and the repeat count was set at 5 with a dynamic repeat time at 30 s. Collision-induced dissociation (CID) was performed with an isolation width of 2 Da. The collision energy was set to 35%. An external calibration for mass accuracy was carried out before the analysis. The measured masses were within 5ppm of the theoretical masses. The data analysis was achieved using XCalibur softwarev2.0.7 (Thermo Fisher Scientific).

### 3.4. Data Processing

The data analysis was processed using Thermo Xcaliber 2.1 workstation (Thermo Fisher Scientific) for peak detection and peak alignment, the raw data were processed by the computer-based NMDF approach.

**Table 2:** Solvent gradient program of UPLC analysis.

Time (min)	Flow (mL/min)	A (%)	B (%)
0	0.300	99.0	1.0
1.0	0.300	99.0	1.0
12	0.300	91.0	9.0
17	0.300	88.0	12.0
17.5	0.300	86.5	13.5
23	0.300	86.5	13.5
33	0.300	70	30.0
35	0.300	60	40.0
50	0.300	35	65.0
52	0.300	35	65.0
55	0.300	1.0	99.0

## 4. Conclusions

In this study, a rapid, sensitive and reliable UPLC-ESI-LTQ-Orbitrap-MS method was established for screening and identifying the chemical constituents of WZYZW in both the positive and negative ion modes. Based on the chromatographic and spectrometric data, and referring to the literature, we were able to tentatively characterize 109 compounds, including organic acids, flavonoids, phenylpropanoids, alkaloids and terpenoids. In all 14 ingredients from *Fructus* *Lych* were identified, 11 ingredients from *Semen Cuscutae* (fried), 35 ingredients from *Fructus Rubi*, 37 ingredients from *Fructus Schisandrae chinensis* (steamed), and 21 ingredients from *Semen Plantaginis* (fried with salt). Our results broadens the chemical knowledge of WZYZW, which should be helpful for the quality control of WZYZW, and for further research on the pharmacokinetic studies and the health and medical properties of WZYZW. Moreover, with the successful application of the UPLC-ESI-LTQ-Orbitrap-MS to characterizing the constituents of WZYZW, it is suggest that this method offer a rapid, sensitive and high throughput methodology for the identification of constituents of TCM prescriptions and herbal medicines.
